# Machine learning to predict effective reaction rates in 3D porous media from pore structural features

**DOI:** 10.1038/s41598-022-09495-0

**Published:** 2022-03-31

**Authors:** Min Liu, Beomjin Kwon, Peter K. Kang

**Affiliations:** 1 Department of Earth and Environmental Sciences, University of Minnesota, Minneapolis, USA; 2 Saint Anthony Falls Laboratory, University of Minnesota, Minneapolis, USA; 3 School for Engineering of Matter, Transport and Energy, Arizona State University, Phoenix, USA

**Keywords:** Biogeochemistry, Environmental sciences, Hydrology, Chemical engineering, Applied physics

## Abstract

Large discrepancies between well-mixed reaction rates and effective reactions rates estimated under fluid flow conditions have been a major issue for predicting reactive transport in porous media systems. In this study, we introduce a framework that accurately predicts effective reaction rates directly from pore structural features by combining 3D pore-scale numerical simulations with machine learning (ML). We first perform pore-scale reactive transport simulations with fluid–solid reactions in hundreds of porous media and calculate effective reaction rates from pore-scale concentration fields. We then train a Random Forests model with 11 pore structural features and effective reaction rates to quantify the importance of structural features in determining effective reaction rates. Based on the importance information, we train artificial neural networks with varying number of features and demonstrate that effective reaction rates can be accurately predicted with only three pore structural features, which are specific surface, pore sphericity, and coordination number. Finally, global sensitivity analyses using the ML model elucidates how the three structural features affect effective reaction rates. The proposed framework enables accurate predictions of effective reaction rates directly from a few measurable pore structural features, and the framework is readily applicable to a wide range of applications involving porous media flows.

## Introduction

Predicting reactive transport in porous media is critical for a wide range of natural processes as well as energy and environmental applications, including geothermal energy recovery^1,2^, subsurface contaminant transport^3–5^, CO_2_ and H_2_ geological storage^6–9^, spent nuclear fuel disposal^10–12^, and water filtration^13,14^. Reaction rate is a key input parameter to reactive transport modeling, which strongly affects prediction results. A key challenge arises from the fact that effective (apparent) reaction rates depend not only on intrinsic chemical properties but also on pore structure and fluid flow conditions^15–17^. This is because reactive transport in porous media is a strongly coupled process involving complex fluid flow, solute transport, and chemical reactions. Indeed, significant discrepancies between reaction rates measured from well-mixed reactors and effective reaction rates measured from column experiments and field observations have been reported^18–21^.

The discrepancies between well-mixed reaction rates and effective reaction rates are known to be caused by both geochemical and physical heterogeneities of porous media systems^19,21–24^. Geochemical heterogeneity originates from the variety of minerals and complexity in chemical reactions^25–31^, while physical heterogeneity is caused by the structural heterogeneity of porous media, which controls fluid flow and mass transfer^22,32–41^. In particular, pore structural heterogeneity is shown to exert dominant control over fluid mixing and homogeneous reaction rates^42–44^, and also shown to control porosity and permeability evolution induced by heterogeneous reactions (i.e., dissolution and precipitation)^17,34,45–50^. Yet, the quantitative relationships between pore structural features (e.g., tortuosity, coordination number) that characterize pore structural heterogeneity and the effective reaction rates are still elusive. This limits our fundamental understanding and predictive capability of reactive transport processes in porous media.

To uncover the quantitative relationship between pore structural features and effective reaction rates in porous media, detailed representations of pore structures and reliable pore-scale modeling methods are needed. Recent advances in pore-scale imaging and modeling techniques enabled the accurate acquisition of pore structural information^51–54^ and high-fidelity pore-scale simulation of reactive transport^22,32,33,38,51–55^. However, these pore-scale direct numerical simulation methods are computationally demanding, which limits most studies to focus on a few porous media samples. Hence, results are often specific to the studied geometries, while pore geometrical complexity is enormously diverse^56,57^.

Machine learning (ML) methods are considered as a powerful alternative to time-consuming numerical simulations while maintaining the accuracy of pore-scale direct numerical simulations^55,58–62^. Recently, deep learning frameworks based on neural networks have been successfully applied to make rapid predictions of the physical properties of porous media, including permeability, porosity, and specific surface area^55,59,61,63–65^. However, ML models have rarely been applied to reactive transport problems^66,67^, and have not yet been used to predict reaction rates in porous media. Furthermore, these previous ML-based investigations of porous media are mostly based on a single ML algorithm. Combinations of different ML algorithms, such as Random Forests (RF) and neural networks, have been used for predictive diagnostics with great success in medical and bioinformatics studies, where each individual algorithm is able to contribute its own strengths toward solving a specific problem^68,69^. Yet, combinations of ML models have rarely been used in reactive transport studies.

We propose an ML-based framework that characterizes the quantitative link between pore structural features and effective surface reaction rates. The pore structural features extracted from hundreds of porous media and effective reaction rates estimated from pore-scale simulations are used as the input data to train ML models. We first train an RF learning model to estimate the importance of pore structural features in determining the reaction rates. Based on this importance information, we then train an artificial neural network (ANN) model and accurately predict effective reaction rates with only three pore structural features. We demonstrate the developed framework in different flow (Pe) and reaction (Da) regimes. Finally, using the ANN model, the effects of the key pore structural features on effective reaction rates are comprehensively evaluated through global sensitivity analyses, and the physical relevance of the results is discussed.

## Methods

In this section, we present the pore-scale numerical method for building the training data, and the ML methods for estimating pore structural features’ importance and predicting the effective reaction rates.

### Building training data with pore-scale reactive transport modeling

We perform a large number of pore-scale reactive transport simulations in an ensemble of 3D porous media structures obtained from an open-source database^64^. Rabbani et al.^64^ generated a large set of porous structures through the texture transformation and porosity manipulation of 60 measured tomographic images. Each sample has a size of 256^3^ voxels, and dimensionless units are used to estimate the physical features of porous media. For each porous media, Rabbani et al.^64^ quantified various pore structural features including 11 of the most common single-value pore structural features. They are specific surface, pore sphericity, coordination number, throat radius, pore radius, tortuosity, pore-throat ratio, grain radius, pore density, grain sphericity, and throat length.

We apply a previously verified 3D pore-scale reactive transport model^70–72^ to calculate effective surface reaction rates in the ensemble of porous media. In this model, fluid flow, solute transport, and chemical reactions are solved. We consider incompressible fluid flow at low Reynolds numbers and solve the continuity equation and the Stokes equation^73^,

1 $$\nabla \cdot \underline{v}=0,$$

2 $$\nabla \text{p}=\upmu {\nabla }^{2}\underline{v},$$

where $\underline{v}$ is the fluid velocity vector, $\text{p}$ is the pressure, and $\upmu$ is the dynamic viscosity. The governing equations of fluid flow are solved by the discrete Boltzmann equation based on the D3Q19 scheme^29^. A no-slip boundary condition is applied via the bounce-back rule at the fluid–solid interface. Details on the applied lattice Boltzmann method can be found in Mostaghimi et al.^34^.

The advection–diffusion equation is then solved to consider the transport of reactive solutes through pore spaces^74^,

3 $$\frac{\partial C}{\partial t}+\left(\underline{v}\cdot \nabla \right)C=\nabla \cdot \left(D\nabla C\right),$$

where $C$ is the local solute concentration, $t$ is the time and $D$ is the molecular diffusion coefficient. To study different flow regimes, we consider three different Péclet (Pe) numbers that cover typical flow conditions in porous media: Pe = 0.1, 1, 10. Pe is defined as $\frac{{U}_{av}L}{D}$ where ${U}_{av}$ is the average velocity and $L$ is the characteristic length that is calculated via $\pi /{s}_{A}$ where ${s}_{A}$ is the specific surface area^34^.

For fluid–solid reactions in the porous media, we consider a bimolecular heterogeneous reaction which can be expressed as a generic equation,

4 $$\text{A}\left(\text{aq}\right)+\text{B}\left(\text{s}\right)=\text{C},$$

where A is chemical species in aqueous solutions, B is the reactant in solid phase and C is the product that can be either in aqueous phase (dissolution) or solid phase (adsorption). This equation has been reported to be adequate for describing chemical reactions in various systems and applications^75–77^. We implement the bimolecular heterogeneous reaction by applying irreversible first-order reaction kinetics as the boundary condition at fluid–solid interfaces via,

5 $$D\frac{\partial C}{\partial \underline{n}}={-k}_{r}C,$$

where ${k}_{r}$ represents the intrinsic reaction rate constant, and $\underline{n}$ denotes the unit normal vector to the solid surface^46^. We then solve for steady-state concentration fields. The modeling framework is applicable to various types of surface reactions by using relevant reaction rate constants. In this study, we consider calcite dissolution and salt ion adsorption scenarios, where ${k}_{r}$ is set as $1.08\times {10}^{-7} [\text{m s}^{-1}]$^51,70^ and $1.0\times {10}^{-6} [\text{m s}^{-1}]$^78^, respectively. The dissolution rate constant for calcite is measured in CO_2_-saturated water in batch reactors at 323 K and at 10 MPa^51^, and the salt ion adsorption rate constant is measured in a carbon membrane capacitive deionization cell with a potential of 1.2 V at pH 7.0^78^. We consider reactive transport of ${\text{H}}^{+}$ and ${\text{Na}}^{+}$ for calcite dissolution and salt ion adsorption scenarios, respectively. We use Damköhler numbers $\left(\text{Da}=\frac{{k}_{r}}{{U}_{av}}\right)$ to describe the reaction rate relative to the mass transfer rate by advection^79^, and use Da_II_ = Pe $\text{Da}=\frac{{k}_{r}L}{D}$ to compare the reaction rate to the mass transfer rate by molecular diffusion^51,80^. The diffusion coefficient of ${\text{H}}^{+}$, ${D}_{{H}^{+}}$, is $1.0\times {10}^{-9} [{\text{m}}^{2} \,{\text{s}}^{-1}]$ and ${D}_{{\text{Na}}^{+}}$ is $1.3\times {10}^{-9} {[\text{m}}^{2}\, {\text{s}}^{-1}]$^81^, and the corresponding Da_II_ values are 0.03 and 0.27. Note that Da is independent of initial concentration because the reaction follows the first-order reaction. Thus, the studied system is determined by Pe and Da (or Da_II_).

**Figure 1:**
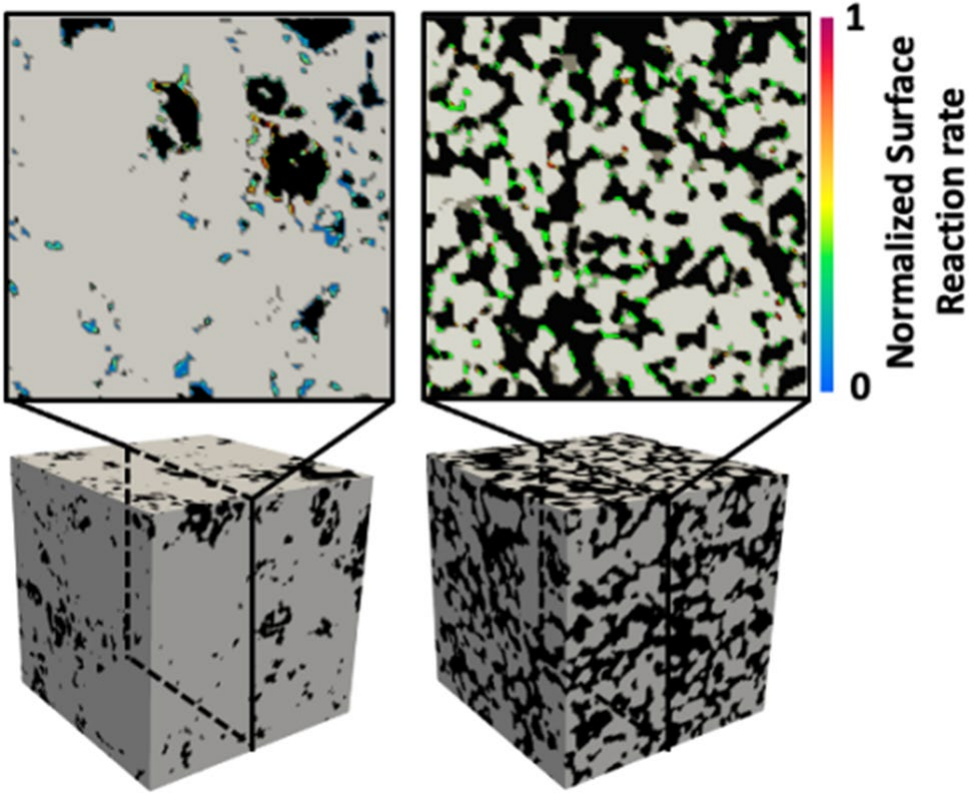
The normalized local surface reaction rates shown in 2D slices (top) of two different 3D porous media (bottom).

From the steady-state concentration fields, we directly estimate the local reaction rate at each interfacial grid (Fig. 1) and average over all interfacial grids to obtain effective reaction rates. The effective surface reaction rate can be defined as^22^,

6 $${R}_{\text{eff}}=\frac{{\sum }_{1}^{N}{k}_{r}{(C}_{i}-{C}_{s})}{N},$$

where $N$ is the total number of voxels at fluid–solid interfaces and ${C}_{i}$ is the steady-state concentration of *i*-th voxel at fluid–solid interfaces. We define the normalized effective reaction rate as, ${R}_{\text{norm}}=\frac{{R}_{\text{eff}}}{{k}_{r}{(C}_{\text{in}}{-C}_{\text{s}})}$, where ${C}_{\text{in}}$ is the injection concentration. ${R}_{\text{norm}}$ quantifies the discrepancy between the effective reaction rate and the well-mixed reaction rate for ${C=C}_{\text{in}}$.

### Machine learning methods

**Figure 2:**
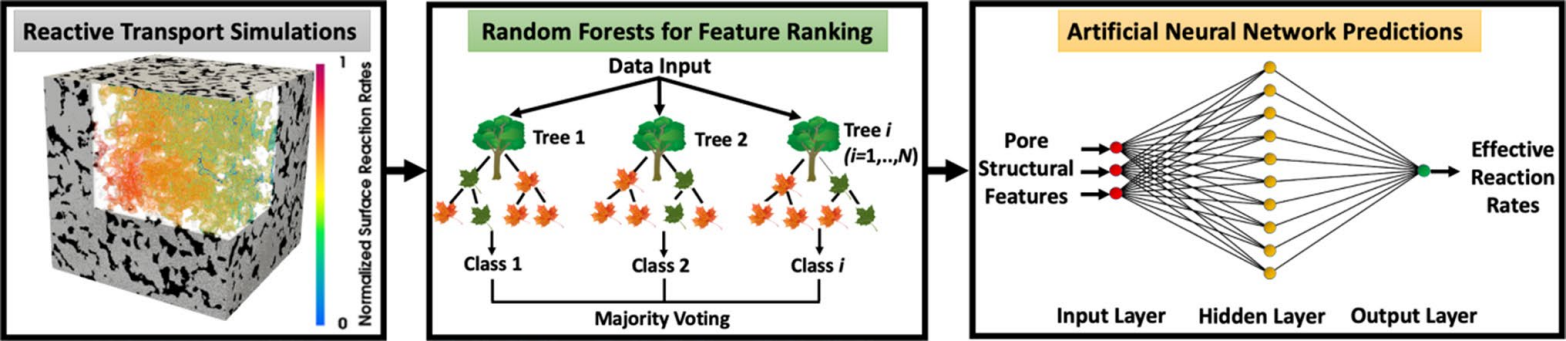
The schematic of the ML-based framework that combines 3D pore-scale reactive transport, Random Forests (RF), and Artificial Neural Network (ANN) models.

The pore structures of porous media can be characterized by multiple features, such as specific surface, tortuosity, and pore radius. We use the 11 single-value pore structural features and effective reaction rates calculated from pore-scale simulations to train a two-step ML framework (Fig. 2), aiming to identify the key pore structural features that control effective reaction rates. We combine RF and ANN models to quantify the importance of pore structural features and to predict effective reaction rates directly from a few key pore structural features. In comparison with neural networks, RF is less computationally expensive and can effectively estimate the importance scores of input features, i.e., RF is a useful algorithm for feature importance ranking^82,83^. In spite of the higher computational cost for training, the ANN offers better model accuracy and performance, if the model is well optimized. Thus, we use RF to estimate feature importance, then use that importance information to optimize ANN training. Feature selection for ML models can help reduce redundant data, minimize overfitting, and improve model accuracy by removing unnecessary data. Further, ML algorithms with fewer features can be trained faster^84,85^.

RF learning algorithm creates multiple decision trees on input data and then selects the mean predictions of each decision tree as the best solution^82,83^. We use the data of 11 pore structural features and corresponding effective reaction rates as input to train a bagged ensemble of decision trees to estimate the importance value for each pore structural feature. The pore structural importance values are estimated by permuting out-of-bag observations among the decision trees, which are calculated as the difference between the benchmark/initial estimations and the one from the permuted predictions^82,83^. The important hyperparameters used in the RF model training are the maximal number of decision splits (299), minimum parent size (10), minimum number of leaf node observations (1), and depth of tree (9). The hyperparameters are optimized to maximize R^2^ values and to ensure enough splits and tree depth.

ANN model applies a learning algorithm for nonlinear statistical data modeling by mimicking the way nerve cells work in the human brain, and the model is particularly efficient in implicitly estimating complex nonlinear relationships between input features and target predictions^86^. We choose a single layer feed-forward neural network consisting of an input layer, hidden layer, and output layer^87–89^. The input is the pore structural features, and effective reaction rates from pore-scale simulations are the target predictions. The hidden layer consists of 10 neurons, where multiple functions are applied for data transformation. The neurons learn about the data and then send it to the output layer. Bayesian Regularization is used as the training algorithm in ANN, which is efficient for training small-size datasets with noises. We first train the ANN with the 11 pore structural features, and then reduce the number of input features based on the importance values obtained from the RF model.

## Results and discussion

In this section, we first present results with an RF learning model that ranks the importance of each pore structural feature in predicting effective reaction rates. Then, we combine the RF importance ranking result with the ANN model to identify the most critical pore structural features for predicting effective reaction rates. We start with the Pe = 0.1 and Da_II_ = 0.03 case and expand to different Pe and Da regimes. Finally, we conduct global sensitivity analyses with the validated ANN model and discuss the results.

### Importance ranking of pore structural features

**Figure 3:**
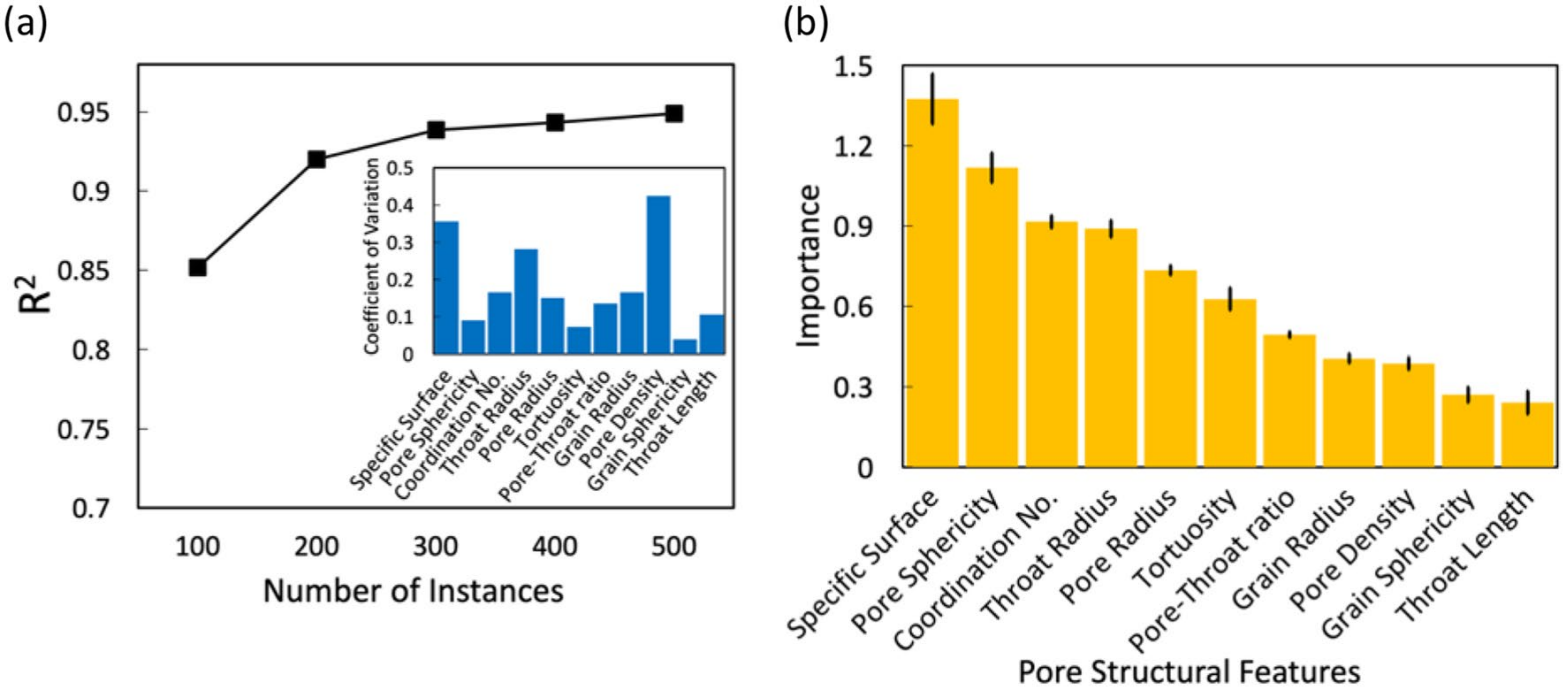
(**a**) The R^2^ for importance estimations as a function of the number of instances. Inset: the coefficient of variations of 11 pore structural features; (**b**) the importance ranking of 11 pore structural features estimated by RF model trained with 300 instances. The error bars represent ± standard error which is obtained from fivefold cross-validations.

For ML predictions, there is generally a trade-off between data preparation cost and model accuracy. ML models often show better performance with more datasets or instances for predictions, though it depends on the particular dataset^62,90^. To test this, we train the RF learning model with pore-scale simulation results of 100, 200, 300, 400, and 500 instances (i.e., the number of porous media samples) to determine how many simulation results are needed to achieve adequate accuracy of the learning model. We use the coefficient of determination, R^2^, to measure the accuracy of the ML models^91^. The black square data points in Fig. 3a show the R^2^ values of RF models as a function of the number of instances used in training. The R^2^ increases as the number of instances increases, but the increase is relatively minimal beyond 300. The R^2^ of the model with 300 training instances is 0.938, which is comparable to that with 500 instances (R^2^ = 0.946). Hence, we train the RF model with 300 instances and estimate the importance of 11 pore structural features.

The RF successfully ranked the importance of each feature, and the results are validated with the fivefold cross-validations. The importance rankings are shown in Fig. 3b, and the importance of each feature is estimated from the increase in the prediction errors after permuting the feature in the datasets^82,83^. The error bars are obtained from fivefold cross-validation, which is commonly used to test the performance of the ML model and detect overfitting^92^. The short error bars indicate that there is small uncertainty in the predicted importance values and the average values of the importance are reliable^82,83^.

Because the importance ranking can be affected by the variability of feature values^90^, we estimate the coefficient of variation of each feature, which quantifies the variability around its mean value^93^. The inset of Fig. 3a shows the coefficient of variation of each pore structural feature. It is worth noting that pore sphericity and tortuosity have lower variability than most of the other features, while they are estimated as the second and sixth most important among the 11 features. There is no noticeable correlation between the feature importance and coefficient of variation, and this indicates that the variability of feature values in the input data is large enough to evaluate the importance of pore structural features.

### Predicting effective reaction rates from key pore structural features

**Figure 4:**
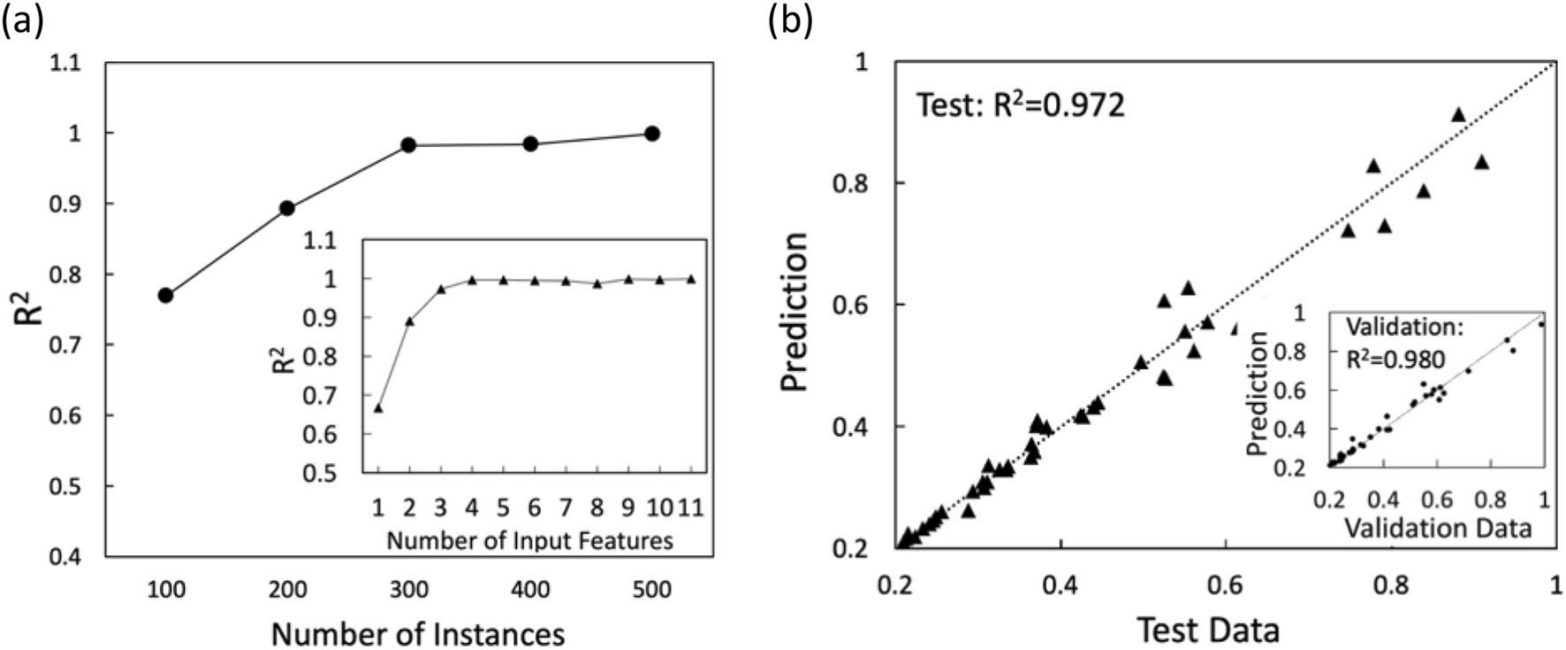
(**a**) ANN predictions (R^2^) as a function of the number of instances. Inset: the impact of the number of input features on ANN predictions using 300 instances. Features are removed one by one starting from the least important feature; (**b**) the testing and validation performance (inset) of ANN predictions using the three pores structural features from 300 instances. The values represent normalized reaction rates.

Based on the importance information, we now train the ANN model with a varying number of features to identify the key features for predicting effective reaction rates. Figure 4a shows the R^2^ values for ANN predictions using 11 pore structural features. When the number of instances used for training is larger than 300, highly accurate and stable predictions are achieved, which suggests 300 instances (R^2^ = 0.972) are also sufficient for establishing an accurate ANN model. The importance estimation of pore structural features by RF provides the basis for identifying key features^94–96^. To identify the most critical pore structural features for ANN predictions, we train the ANN using 300 instances with 11 features, and then reduce the number of input features one by one, removing the features with the lowest importance value. The importance ranking from the most important feature to the least important feature are the following: specific surface, pore sphericity, coordination number, throat radius, pore radius, tortuosity, pore-throat ratio, grain radius, pore density, grain sphericity, throat length. The inset in Fig. 4a shows no significant variations in R^2^ values when the ANN model is trained with three or more input features. When using one or two features for training, the R^2^ values are much lower, indicating the necessity of using the three most important pore structural features for achieving accurate predictions^64,97–99^. This implies that the other pore structural features are highly related to these three features or have limited contributions to effective reaction rates, which can be confirmed from the pair-wise correlations between the 11 features (see the Supplementary Document). For example, throat radius shows a high correlation with pore sphericity, and tortuosity shows a high correlation with coordination number.

Specific surface, pore sphericity, and coordination number are identified as the three most important pore structural features. Specific surface area has the highest importance, which is defined as the ratio of total surface area to bulk volume^64^. This result is intuitive because the specific surface is directly linked to the reactive surface area^100,101^. Pore sphericity is a shape factor describing the smoothness of reactive surface^97,98^, and it will affect the efficiency of mass transfer from the fluid to solid surfaces. Coordination number measures the average number of throats connected to a pore and describes the average connectivity of the pore space, which will govern the overall accessibility of reactive surface^99^.

We use these three features from 300 instances as inputs to train the ANN model that predicts effective reactions rates. We use 70% of the data for training, 15% for validating, and 15% for testing the model. The testing data is independent of the training and validation data, which is applied to measure the performance of the trained ANN model. The prediction performance of the ANN model is shown in Fig. 4b. The validation R^2^ is 0.980, and the testing R^2^ is 0.972, indicating the ANN model provides good performance in predicting effective reaction rates with only three pore structural features. The marginal discrepancy between the R^2^ values from validation and testing shows the stable performance of the ML model^90,102^.

### The effects of Pe and Da

**Figure 5:**
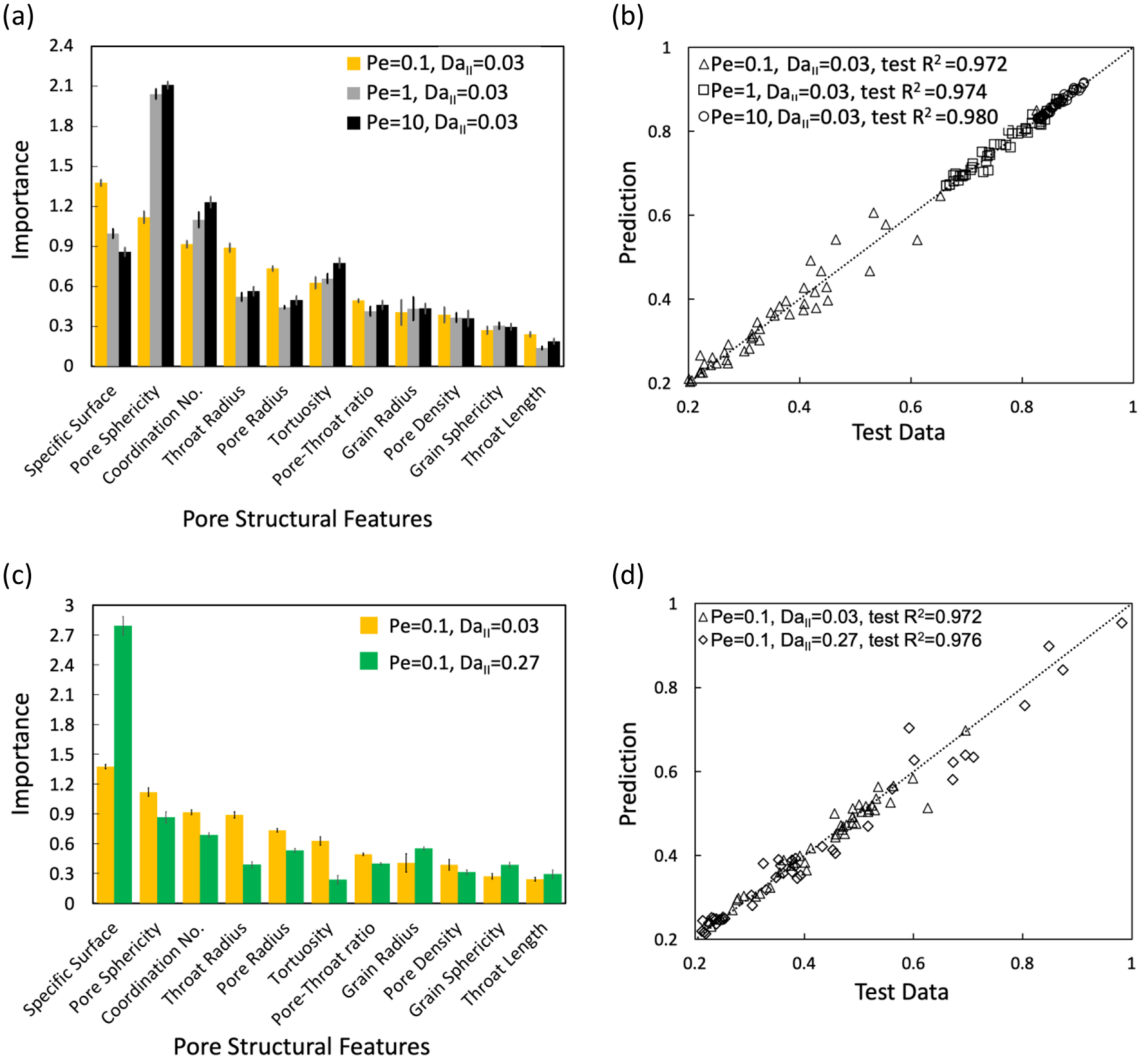
(**a**) Importance estimation of pore structural features at three different Pe (0.1, 1, and 10) with Da_II_ = 0.03 (the corresponding Da values are 0.3, 0.03, 0.003) and (**b**) the corresponding prediction performance of ANN models using the three pore structural features from 300 instances; (**c**) importance estimation of pore structural features at Da_II_ = 0.03 and 0.27 (the corresponding Da values are 0.3, 2.7) with Pe = 0.1 and (**d**) the corresponding prediction performance of ANN models using the three pore structural features.

We extend the developed framework to different flow (Pe) and reaction (Da) regimes. We first present the results under different Pe numbers. Figure 5a shows the results of importance estimation at Pe = 0.1, 1, and 10 with Da_II_ = 0.03. Specific surface, pore sphericity, and coordination number remain the three most important features, though the most important feature becomes pore sphericity at Pe = 1 and 10. At higher Pe, advection is stronger, meaning pore structural features that are sensitive to flow will become more important. Indeed, the importance of pore sphericity and coordination number increases as Pe increases. The pore sphericity measures the shape of fluid-pore interfaces and determines the smoothness of the flow lines in pore space, thereby also the accessibility of the reactive surface area. Therefore, the influence of the pore shape factor on reaction increases as Pe increases. The results show that the three most important features remain the same across Pe numbers, indicating that the three features are the key predictors for effective reaction rates in typical porous media flow conditions. Figure 5b shows the test R^2^ of the ANN predictions at different Pe numbers. The test R^2^ values for ANN predictions at Pe = 0.1, 1 and 10, are calculated as 0.972, 0.974 and 0.980, respectively. The R^2^ values of ANN predictions are high across Pe numbers, showing stable and high-quality predictions across these different flow conditions.

We now extend the framework to a different Da number (Da_II_ = 0.27) with a higher intrinsic reaction constant ($1.1\times {10}^{-6} [\text{m} {\text{s}}^{-1}]$), which is relevant for Capacitive Deionization (CDI), an emerging desalination method^78^. The transport of Na^+^ and its adsorption at the solid surface is considered in the model. As shown in Fig. 5c, the three most important features are the same at both Da_II_ = 0.03 and 0.27 with Pe = 0.1. The increase in reaction constant leads to a large increase in the estimated importance of the specific surface (the first green bar), which indicates that the specific surface plays a much more important role at higher reaction rates. At low flow rates (Pe = 0.1) but high reaction constant, the reaction rate is much larger than the advective mass transport rate, making the surface area play a more dominant role in determining the amount of fluid–solid reaction. Figure 5d shows the ANN predictions with two Da numbers. A high R^2^ value of 0.976 is again achieved at Da_II_ = 0.27, only with the three pore structural features. This result confirms that the ML framework and findings are also valid for different Da regimes.

### Global sensitivity analysis with machine learning

**Figure 6:**
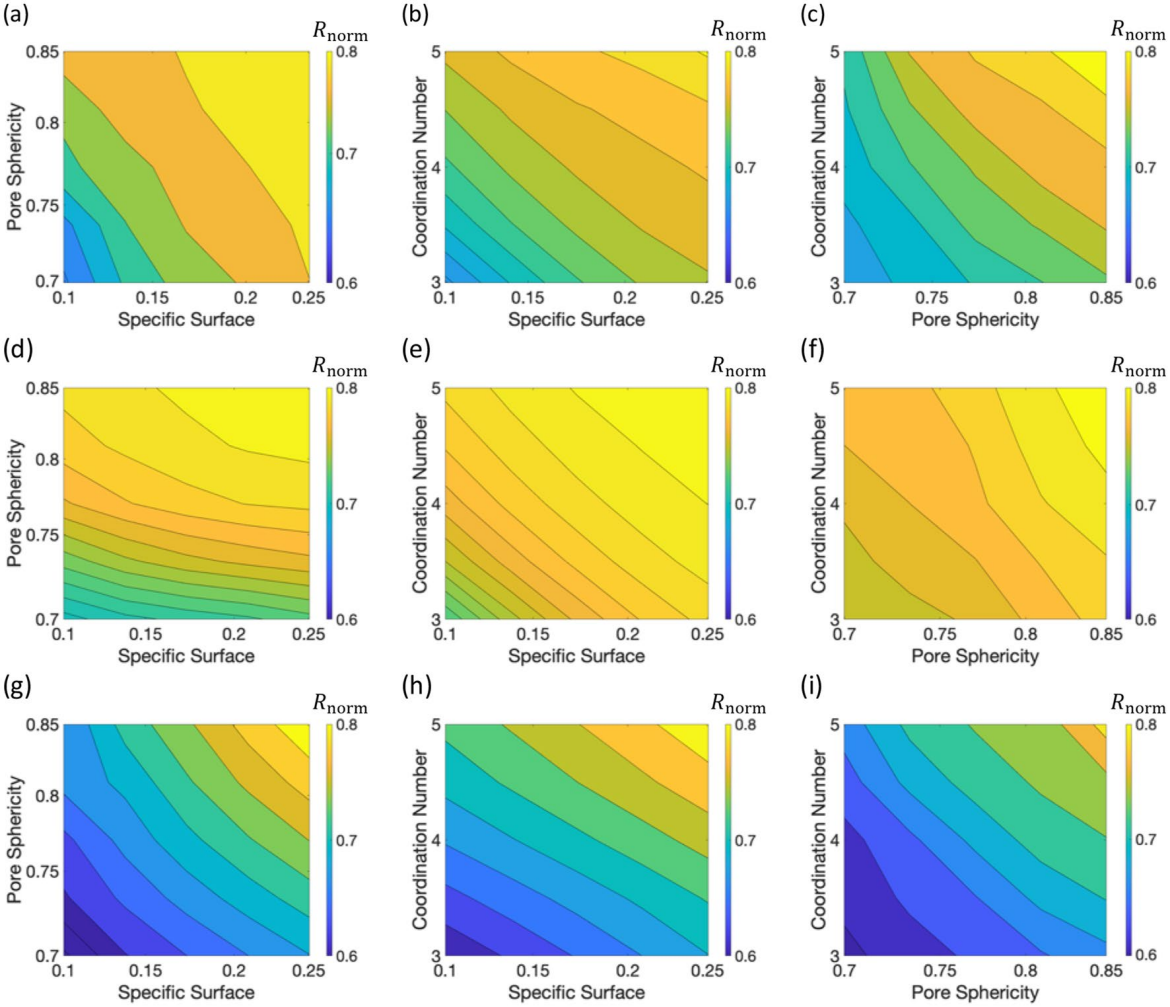
The effect of specific surface, pore sphericity and coordination number on normalized effective reaction rates (${R}_{\text{norm}}$) under different Pe and Da conditions: (**a–c**) at Pe = 0.1, Da = 0.3, Da_II_ = 0.03; (**d–f**) at Pe = 1, Da = 0.03, Da_II_ = 0.03; (**g–i**) at Pe = 0.1, Da = 2.7, Da_II_ = 0.27. The values of coordination number (4.6) in the left column, pore sphericity (0.76) in the middle column, and specific surface (0.13) in the right column are fixed to their average values in the dataset.

We perform global sensitivity analyses using the trained ANN model to elucidate the combined effects of the key pore structural features on effective reaction rates under various flow and reaction conditions. Each row in Fig. 6 shows the effects of pore structural features on normalized effective reaction rates (${R}_{\text{norm}}$) at fixed Pe and Da. For each column, the coordination number, pore sphericity, and specific surface are fixed respectively with their average values in the datasets. This enables us to plot the combined effects of two features on the effective reaction rates. Effective reaction rates expectedly increase with specific surface and pore sphericity, as these two features determine the area and accessibility of pore reactive surface. A larger coordination number also leads to a larger effective reaction rate because a large coordination number implies an enhanced mass transfer between pores^64^.

The overall effects of the three features on effective reaction rates are similar for the explored Pe and Da cases. However, the magnitude of normalized effective reaction rate is sensitive to Pe and Da. At higher Pe but low Da, the system is reaction-limited^21,46^. In this regime, the mass transfer rate is faster than the reaction rate, such that the concentration and reaction rates in the pore space become spatially uniform, leading to the increase in ${R}_{\text{norm}}$ (compare Fig. 6a,b and d–f). We confirmed that the Pe = 10 case leads to a further increase in ${R}_{\text{norm}}$, meaning less discrepancy between the well-mixed reaction rate and effective reaction rate. Figure 6g–i show the ANN predictions with Da = 2.7 at Pe = 0.1, where a large variability in effective reaction rates is estimated. This reaction regime is transport-limited due to weak advection (Pe < 1) and high reaction rate (Da > 1)^33,46^, where the reaction rate is higher than the mass transfer rate by advection. In such regimes, uneven distributions of concentrations and large concentration gradients emerge in the pore space, leading to discrepancies between the well-mixed reaction rate and effective reaction rate. The global sensitivity analyses elucidate the effects of the key pore structural features on effective reaction rates, and the effective reaction rates could be used as input parameters to Darcy-scale reactive transport modeling.

If solid phase alteration is considered, uniform or transitional (between uniform and wormholing) dissolution patterns are most likely to be observed due to the low Da numbers^35,103^. However, it is known that the dissolution patterns depend not only on Pe and Da but also on pore structural heterogeneity^38,46,104^. The ML framework proposed in this study could be extended to predict dissolution regimes from Pe, Da, and pore structural features. However, reactive flow simulations with solid alterations, which is computational very expensive, should be performed to obtain accurate dissolution patterns.

## Conclusions

Numerous environmental applications rely on reactive transport in porous media, but the accurate estimation of reaction rates has been a major challenge, limiting the predictive capability of reactive transport models. This study established a quantitative link between pore structure features and effective surface reaction rates by combining pore-scale simulations with ML algorithms. For the first time, we identified the three key pore structural features that determine effective surface reaction rates. The three features remained as the top critical features for the explored values of Pe and Da, which cover typical flow and reaction regimes in porous media^105,106^. The identified three features indeed capture the key factors that control reactive transport with heterogeneous reactions: specific surface quantifies surface area effect, pore sphericity quantifies pore shape effect, and coordination number quantifies flow/connectivity effect. We also applied this ML-based framework to perform global sensitivity analyses of the input features in determining effective reaction rates. The established ML model served as a surrogate model and enabled us to exhaustively and efficiently evaluate the effects of various system parameters (e.g., pore structures, flow rates, reaction rate constants) on effective reaction rates, which was otherwise not feasible due to the computational limitations. Extending the applicability of the proposed framework to wider ranges of Pe and Da will be an important next step. Also, with a larger dataset with wider Pe and Da values, one may be able to develop a more generic ML model that includes Pe and Da as input parameters.

The presented ML framework can be readily extended to a wide range of geological and environmental applications that involve complex coupled processes. For example, the lifetime of bentonite barriers in geologic repositories of spent nuclear fuel could be efficiently estimated by using clay structural features, temperature, chemical reaction constants, water saturation, and swelling rates as inputs to the ML model training. Further, the framework can be used to not only establish a quantitative link between input variables and target output variables but also to identify optimal values of pore structural features that can maximize the performance of porous materials. For example, by linking the pore structural features to effective corrosion rates, the framework can identify novel corrosion-resistant porous materials with optimized pore structural features that minimize corrosion. The framework can also be naturally extended to optimize other material properties such as mechanical strength and filtration efficiency. In particular, the model could identify optimal membrane properties for maximizing filtration efficiency by considering fiber diameter, hierarchical surface structure, pore size distribution, and surface area as input parameters and the effective water filtration rate as target prediction.

The proposed ML framework also provides an attractive approach for obtaining upscaled model parameters that are physically parameterized with subscale properties. In subsurface applications, the continuum model is often incapable of properly capturing pore-scale effects on Darcy-scale properties such as permeability, dispersion coefficients, and effective reaction rates. The proposed framework will enable us to establish ML-based quantitative correlations between pore-scale information and upscaled parameters. In a future study, the effects of porous media sample size should be further investigated. Studying the sample size effect will require substantial computational resources, but it is an important step for achieving upscaling. In summary, the proposed framework can not only elucidate the key parameters that control various physicochemical processes in porous media systems but also can be extended to improve model predictability and to identify optimal properties of porous materials.

## Supplementary Information

Supplementary Figure S1.

## Data Availability

The training data and trained ML models are all made available open access at https://drive.google.com/drive/u/2/folders/17nTPOjOVslivzZG8u0_-l4gibCV3vzHX.
