# Supplementary material for: Machine learning to predict effective reaction rates in 3D porous media from pore structural features
Source: Sci Rep. 2022 Mar 31;12:5486. doi: 10.1038/s41598-022-09495-0 (PMC8971379; doi:10.1038/s41598-022-09495-0)
Supplement: Supplementary file 1 — Supplementary Figure S1. [file 41598_2022_9495_MOESM1_ESM.docx]

*Scientific Reports*

Supporting Information for

Machine Learning to Predict Effective Reaction Rates in 3D Porous Media from Pore Structural Features

**Min Liu^1,2^, Beomjin Kwon^3^, Peter K. Kang^1,2, *^**

1. Department of Earth and Environmental Sciences, University of Minnesota, Minneapolis, USA

2. Saint Anthony Falls Laboratory, University of Minnesota, Minneapolis, USA

3. School for Engineering of Matter, Transport and Energy, Arizona State University, Phoenix, USA

**Contents of this file**

Total pages: 0

Figure count: 1

Table count: 0

Figure S1 shows the pair-wise correlations between the 11 features that quantify the inter-correlation between the features.


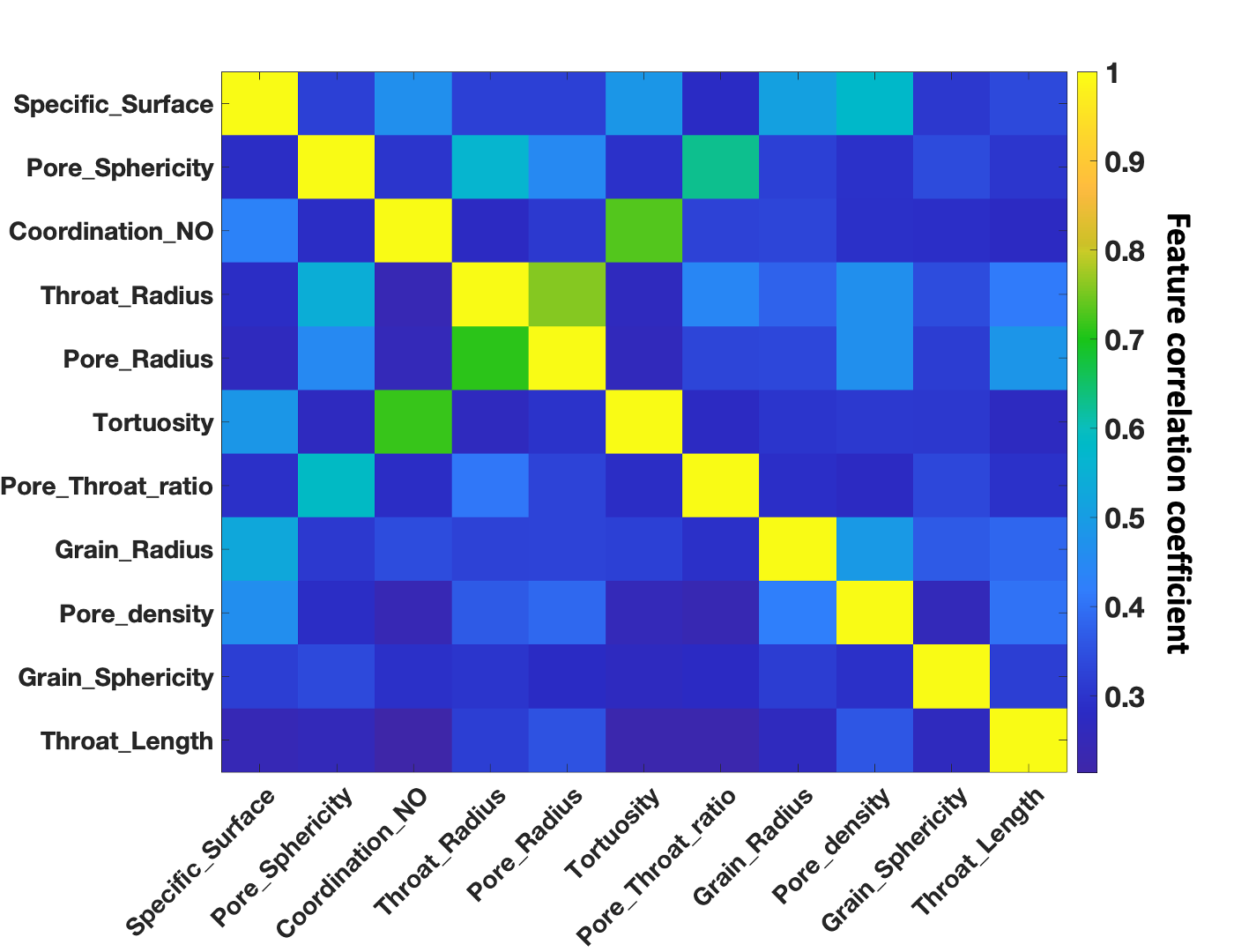


Figure S1 Pair-wise correlation coefficients between 11 pore structural features
